# Persistent Photoluminescence and Mechanoluminescence of a Highly Sensitive Pressure and Temperature Gauge in Combination with a 3D‐Printable Optical Coding Platform

**DOI:** 10.1002/advs.202408686

**Published:** 2024-10-10

**Authors:** Teng Zheng, Jiangcheng Luo, Dengfeng Peng, Liang Peng, Przemysław Woźny, Justyna Barzowska, Mikołaj Kamiński, Sebastian Mahlik, Jan Moszczyński, Kevin Soler‐Carracedo, Fernando Rivera‐López, Hanoch Hemmerich, Marcin Runowski

**Affiliations:** ^1^ School of Information and Electrical Engineering Hangzhou City University Hangzhou 310015 China; ^2^ Key Laboratory of Optoelectronic Devices and Systems of Ministry of Education and Guangdong Province College of Physics and Optoelectronic Engineering Shenzhen University Shenzhen 518060 China; ^3^ Shenzhen Key Laboratory of Intelligent Optical Measurement and Detection Shenzhen University Shenzhen 518060 China; ^4^ Shenzhen Key Laboratory of Photonics and Biophotonics Shenzhen University Shenzhen 518060 China; ^5^ Faculty of Chemistry Adam Mickiewicz University Uniwersytetu Poznańskiego 8 Poznań 61‐614 Poland; ^6^ Institute of Experimental Physics, Faculty of Mathematics Physics and Informatics University of Gdansk Wita Stwosza 57 Gdansk 80‐308 Poland; ^7^ Departamento de Física, IUdEA IMN and MALTA Consolider Team Universidad de La Laguna San Cristóbal de La Laguna Santa Cruz de Tenerife E‐38200 Spain; ^8^ Departamento de Ingeniería Industrial Escuela Superior de Ingeniería y Tecnología Universidad de La Laguna San Cristóbal de La Laguna Santa Cruz de Tenerife E‐38200 Spain

**Keywords:** mechanoluminescence, multifunctional optical sensor, persistent‐luminescent 3D‐printed polymers, pressure sensor, visual manometer

## Abstract

Distinct types of luminescence that are activated by various stimuli in a single material offer exciting developmental opportunities for functional materials. A versatile sensing platform that exhibits photoluminescence (PL), persistent luminescence (PersL), and mechanoluminescence (ML) is introduced, which enables the sensitive detection of temperature, pressure, and force/stress. The developed Sr_2_MgSi_2_O_7_:Eu^2+^/Dy^3+^ material exhibits a linear relationship between ML intensity and force and can be used as an ML stress sensor. Additionally, the bandwidth of the PL emission band and the PL lifetime of this material are remarkably sensitive to temperature, with values of ≈0.05 nm K^−1^ and 1.29%/K, respectively. This study demonstrates PersL pressure sensing for the first time, using long‐lasting (seconds) lifetime as a manometric parameter. The developed material functions as an exceptionally sensitive triple‐mode visual pressure sensor; specifically, it exhibits: i) a sensitivity of ≈−297.4 cm GPa^−1^ (8.11 nm GPa^−1^) in bandshift mode, ii) a sensitivity of ≈272.7 cm^−1^/GPa (14.8 nm GPa^−1^) in bandwidth mode, and iii) a sensitivity of 42%GPa^−1^ in PL‐lifetime mode, which is the highest value reported to date. Notably, anti‐counterfeiting, night‐vision safety‐sign, 8‐bit optical‐coding, and QR‐code applications that exhibit intense PersL are demonstrated by 3D‐printing the studied material in combination with a polymer.

## Graphical abstract

A multifunctional sensing platform of temperature, pressure, and stress is built, which is based on three different types of luminescence, photo‐, persistent‐, and mechano‐luminescence. Combined with the 3D‐printing technique, it also has practical uses like anti‐counterfeiting, night‐vision safety‐sign, and 8‐bit optical‐coding and QR codes.

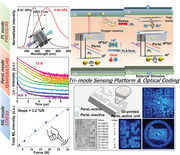

## 1 Introduction

The development of luminescent materials has propelled significant advancements in science and technology.^[^ ^1^ ^]^ Various sources of excitation, including mechanical force, electromagnetic radiation, electrical currents or fields, temperature, or magnetic fields can yield distinct forms of luminescence, including mechanoluminescence (ML),^[^ ^2^ ^]^ photoluminescence (PL),^[^ ^3^, ^4^, ^5^, ^6^, ^7^, ^8^, ^9^ ^]^ electroluminescence (EL),^[^ ^10^ ^]^ and magnetic induced luminescence (MIL).^[^ ^11^ ^]^ The development of multimodal luminescent materials that respond to multiple stimuli has amplified their utility in various applications. For instance, ML manifests as photon emissions when subjected to mechanical forces within solids, including impacts, friction, compression, fractures, grinding, and stretching. As novel smart sensing and imaging materials, ML phosphors have garnered broad attention during the development of artificial skin, flexible electronics, and robotics, as examples. As a pivotal branch of next‐generation sensing systems, stress‐sensing technologies based on ML offer many benefits, e.g., self‐powering, excellent stretchability, biocompatibility, stress/force‐distribution visualization, and remote detection.^[^ ^12^, ^13^, ^14^ ^]^

Persistent luminescence (PersL), as a unique type of luminescence, can produce an afterglow emission that lasts continuously for seconds, hours, or even days after cessation of the excitation source,^[^ ^15^ ^]^ because the incident energy can be stored in PersL‐material traps and then slowly released a long‐lasting light emission. PersL materials are widely used in emergency‐signage, optical‐data‐storage, night‐vision‐surveillance, and in vivo‐bioimaging applications, among others.^[^ ^15^, ^16^, ^17^ ^]^ The development of novel materials for sensing pressure or temperature may involve an efficient strategy that exploits the long lifetimes of afterglow materials. Such long lifetimes are easily measured and are highly affected by temperature and/or pressure, resulting in a highly sensitive kinetics‐based detection method. For instance, Eu^2+^ ion doped M_2_MgSi_2_O_7_ (i.e., alkaline earth akermanite) is among the most common PersL materials, exhibiting long‐lasting PersL for up to 10 h.^[^ ^18^, ^19^, ^20^, ^21^, ^22^, ^23^, ^24^ ^]^

In materials science, pressure, as an essential thermodynamic quantity, can regulate crystal structures, reshape chemical bonds, modify electronic energy‐level structures, change electric/magnetic/light‐related features, and generate new material structures.^[^ ^25^, ^26^, ^27^, ^28^, ^29^ ^]^ For instance, the pressure‐induced polymerization of aromatic molecules such as benzene facilitates the synthesis of highly stable ultrathin and super‐hard ordered carbon nanothreads.^[^ ^30^ ^]^ Various material‐preparation and processing technologies that cannot be completed under ambient conditions, such as the transformation of gaseous hydrogen into its metallic form, are easily accessed under high‐pressure conditions;^[^ ^31^ ^]^ consequently, fast and accurate pressure detection is important in both science and industry. Currently, standard pressure sensors, such as capacitive or piezo‐resistive pressure sensors, require physical contact with the measured object, have long response times, and also exhibit relatively low accuracies and resolutions. Compared to a traditional pressure sensor, a luminescent manometric material benefits from high spatial resolution (sensing in submicron‐sized areas), real‐time monitoring (millisecond response times), and resistance to electromagnetic interference and corrosion, among others. The commonly applied ruby (Al_2_O_3_:Cr^3+^) pressure gauge has several drawbacks, including a relatively low‐pressure sensitivity (d*λ*/d*p* = 0.365 nm GPa^−1^), strong temperature dependence, and strong emission intensity deteriorations with pressure, which hinders the pressure detection in high‐pressure range. Various strategies are currently being developed to enhance the emission signal, including exploiting the exposed nature of 5d electrons (strong crystal‐field effect),^[^ ^32^, ^33^, ^34^ ^]^ Eu^2+^‐Sm^2+^ energy transfer,^[^ ^35^ ^]^ configurational crossover between 4f and 5d states (electron transfer),^[^ ^36^ ^]^ and others.^[^ ^37^, ^38^ ^]^ An excellent method for developing an ultra‐sensitive visual pressure sensor may involve the use of PersL (i.e., the long‐lasting (seconds) lifetime) as a manometric parameter, which, to the best of our knowledge, has not been reported to date.

On the other hand, employing thermally sensitive phosphors as noncontact temperature sensors provides rapid, remote, and sensitive readouts with submicron spatial resolutions that surpass the capabilities of traditional thermometers.^[^ ^39^, ^40^, ^41^, ^42^ ^]^ Boltzmann‐type luminescent thermometers experience bottlenecks involving insufficient relative sensitivity (S_r_) due to the limited energy gaps (≈200–2000 cm^−1^) associated with their thermally coupled levels. Many new strategies for improving this situation have been proposed, including the use of non‐thermally coupled levels, multicenter luminescent materials, highly sensitive 5d levels,^[^ ^43^, ^44^ ^]^ and non‐linear optical effects,^[^ ^45^, ^46^, ^47^ ^]^ among others.^[^ ^40^, ^42^, ^44^, ^48^ ^]^ Moreover, very limited literature on PersL‐based lifetime thermometry, which also provides a powerful strategy for boosting sensitivity, exists.^[^ ^49^, ^50^ ^]^

Herein, we report a multifunctional sensing platform based on a Sr_2_MgSi_2_O_7_ material co‐doped with Eu^2+^ and Dy^3+^ that exhibits three types of luminescence: photo‐, mechano‐, and persistent luminescence, thereby enabling highly sensitive remote temperature, pressure, and stress sensing. The developed Sr_2_MgSi_2_O_7_:Eu^2+^/Dy^3+^ (SMSO) material can be used as an ML stress sensor in the 3–30 N range owing to the linear dependence between ML intensity and force. Moreover, the FWHM and PL lifetime of the developed SMSO material responds strongly to temperature, leading to high sensitivities of ≈0.05 nm K^−1^ and 1.29%K^−1^, respectively. Most importantly, this is the first report that describes the use of PersL for pressure sensing and its long‐lasting (s) lifetime as a manometric parameter. Accordingly, the developed SMSO material can be used as a triple‐mode manometer that exhibits superior sensitivity, with bandshift, bandwidth, and lifetime modes exhibiting sensitivities of ≈−297.4 cm^−1^/GPa (8.11 nm GPa^−1^), ≈272.7 cm^−1^/GPa (14.8 nm GPa^−1^), and ≈42%GPa^−1^, respectively. Notably, high pressure can be visually detected with the naked eye owing to the significant PL color change. Thanks to the successful optimization of the selected material in order to obtain the most efficient PersL, the polymers doped with the SMSO material were 3D‐printed and showed anti‐counterfeiting, night‐vision safety‐sign, 8‐bit optical‐coding, and QR‐code applications.

## 2 Results and Discussion

### 2.1 Strategy and Design

**Figure 1:**
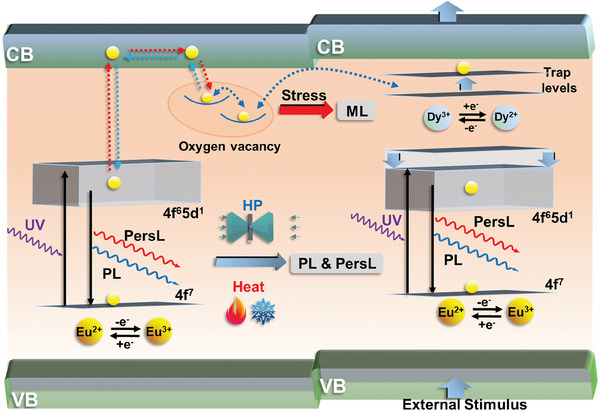
Schematic depicting the proposed, simplified, energy‐migration mechanisms within the developed SMSO:Eu^2+^/Dy^3+^ sensing platform (i.e., PL dual‐mode for sensing temperature and pressure, PersL mode for detecting pressure, and ML mode sensing stress.

We first introduce and discuss the mechanisms governing the PL, PersL, and ML processes in the developed sensing platforms based on the synthesized Sr_2_MgSi_2_O_7_:Eu^2+^/Dy^3+^ material and how pressure, temperature, and stress affect these phenomena. **Figure** **1** shows possible mechanisms that govern the ML and PersL processes in the developed multi‐functional sensing platform.^[^ ^51^, ^52^ ^]^ The deep‐trapped electrons stored in the oxygen vacancies and trap levels of Dy^3+/2+^ are released when subjected to a force (stress) stimulus during the electron‐relaxation process, and follow a reverse route to finally emit ML, whose intensity is highly sensitive to the force (stress) applied to the material, thereby enabling stress to be accurately detected. The PL and PersL mechanisms are explained as follows: Under ambient conditions, the UV excitation energy leads to an electronic transition from the 4f^7^ ground level to the 4f^6^5d^1^ excited level. Some of the electrons immediately return to the 4f^7^ level and emit light (PL). Because the lowest 5d level of the Eu^2+^ ion is close to the conduction band (CB), some electrons are released from the 5d level to the CB; these electrons can be trapped and stored in trap levels at different depths (i.e., in oxygen vacancies and Dy^3+^/Dy^2+^ traps). Dy^3+^ dopants act as trap‐creating ions, which are capable of significantly prolonging afterglow luminescence. PersL is produced by following the reverse route. Electrons are released from the trap in the presence of sufficient thermal energy when the excitation source is extinguished (UV light off). Some of the trapped electrons located in shallow traps escape more quickly to the CB, then to the 4f^6^5d^1^ level, and finally to the ground 4f^7^ level of Eu^2+^, accompanied by the emission of light at ≈473 nm (radiative transition process). Deep‐trapped electrons are subsequently gradually released; this process is affected by the deep‐trap density leading to long‐lasting PersL. The d–f emission from Eu^2+^ is highly efficient, and its wavelength depends strongly on the host lattice and local environment. Notably, variations in the crystal field and the nephelauxetic effect caused by pressure affect the interactions between the 5d electrons of Eu^2+^ and the oxygen ligands. On one hand, the shorter bond length is caused by high‐pressure compression, leading to the enhanced covalent nature between lanthanide‐oxygen bonds, as well as the decrease of the free‐ion parameter and the energy gap between configurations.^[^ ^32^, ^38^, ^53^ ^]^ On the other hand, a stronger energy level splitting is caused by the crystal field effect.^[^ ^35^, ^54^ ^]^ However, a rise in temperature causes the material to expand, thereby increasing the distance between ions, which decreases the strength of the crystal field and the nephelauxetic effect. The energy‐level structure is consequently modified, and the trap‐level release process may also vary. Temperature/pressure changes generally impact the spectroscopic characteristics of a divalent‐lanthanide‐ion‐doped material, including its spectral shift (redshift or blueshift), bandwidth, lifetime (shortening or lengthening), and band intensity ratio, among others.

### 2.2 Properties Under Ambient Conditions

The properties of the Sr_2_MgSi_2_O_7_:2%Eu^2+^/2%Dy^3+^ sample (denoted as SMSO in the following text) were characterized under ambient conditions, including its XRD pattern, PL emission spectrum, PL decay curve, SEM image, EDX spectra, and elemental maps, and are presented in Figures S1 and S2 (Supporting Information) in the Supporting Information (SI). Figure S1a (Supporting Information) depicts the powder XRD pattern of the synthesized material, which is consistent with the standard reference pattern of Sr_2_MgSi_2_O_7_ (PDF #75‐1736). The PL excitation and emission spectra of the sample synthesized under ambient conditions are shown in Figure S1b,c (Supporting Information). The PL excitation spectrum measured at λ_em_ = 473 nm consists of two intense broad bands in the 270–450 nm range, attributed to 4f^7^→4f^6^5d^1^ (f‐d) transition of Eu^2+^, with maxima at 302 and 355 nm. Meanwhile, the emission band, which is attributable to the 4f^6^5d^1^→4f^7^ (d–f) transition, is located in the 410–625 nm range and centered at 473 nm. Figure S1 (Supporting Information)d displays the PL decay curve of SMSO, which shows a lifetime of ≈0.22 µs. Figure S2a (Supporting Information) displays an SEM image of the prepared sample that shows micron‐sized irregularly shaped aggregated particles. The EDX spectra and elemental maps shown in Figure S2b–h (Supporting Information) confirm the presence of uniformly distributed Dy, Eu, Sr, Si, O, and Mg in the synthesized micron‐sized particles.

### 2.3 ML Stress Sensor

**Figure 2:**
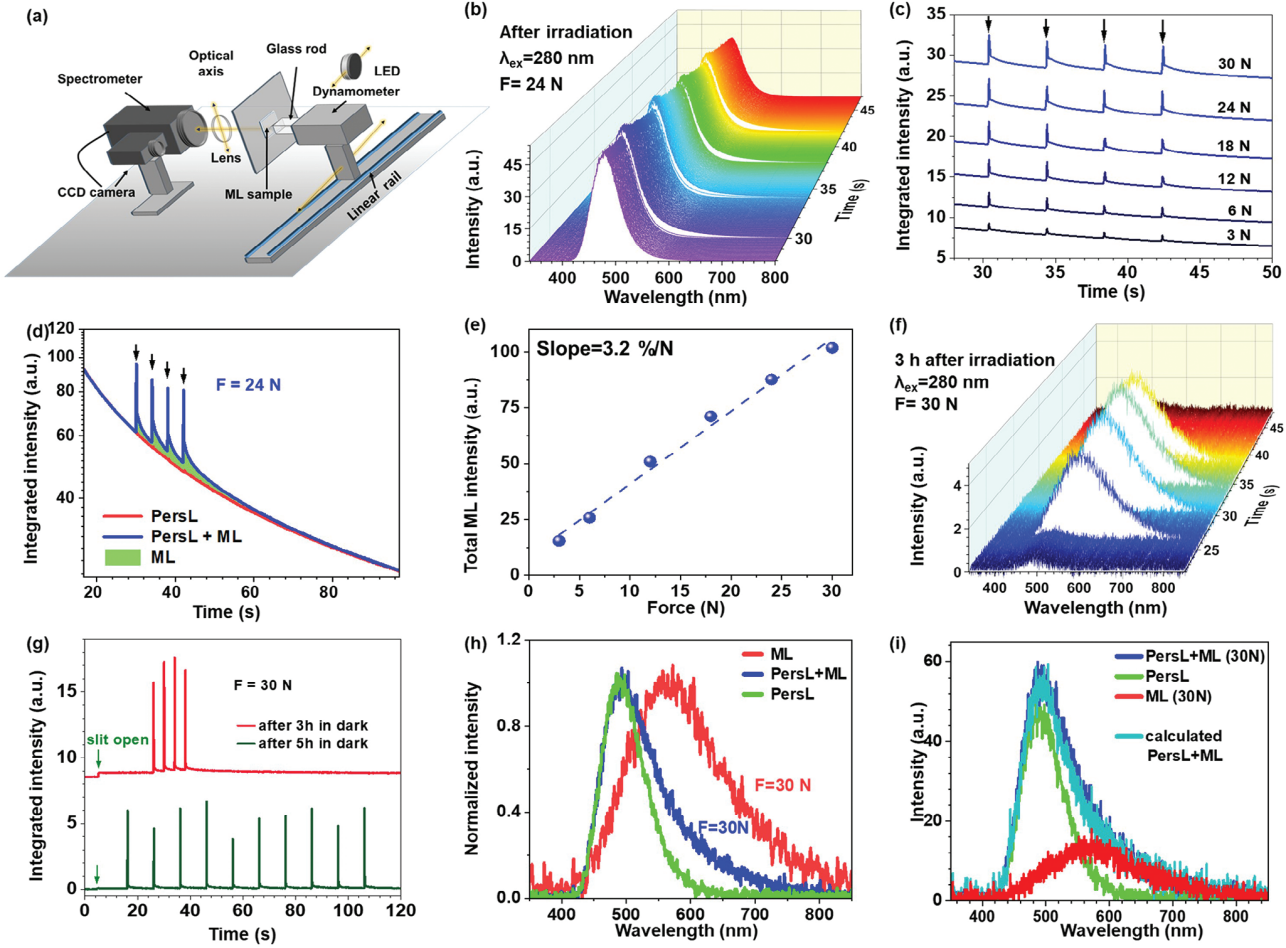
a) Scheme showing the experimental setup used in the ML experiments involving the SMSO material. b) Set of emission spectra taken immediately at the end of the irradiation stage. c) Time dependences of the integrated emission signal (PersL+ML) acquired at different applied force values; the arrows indicate moments when the rod was dragged across the sample plate. d) An example comparison of PersL (green trace) against PersL+ML (blue trace) at 24 N illustrates how the total ML was calculated (total ML, which is equal to the integral of the ML signal over time, is represented by the pink area). e) The dependence of total ML on applied force (the dashed line) is the result of fitting experimental data to a linear function. f) The set of emission spectra was taken 3 h after the end of the irradiation stage. g) Relationship between integrated emission intensity and time acquired 3 h (upper) and 5 h (bottom) after the irradiation step. h) Normalized spectra: uncorrected PersL (green), induced with a mechanical load of 30 N before the PersL signal was quenched (blue), and after PersL had been quenched (purple). i) Comparing the experimental PersL+ML (blue) spectrum with the calculated linear combination (orange) of the ML (purple) and PersL (green) spectra.

A simplified schematic of the setup used to examine the ML properties of the SMSO sample is shown in **Figure** **2**a, with experimental details provided in the SI. Prior to any PersL or ML experiment, the sample was placed in a friction‐induced ML setup and maintained in the dark until the PersL signal had faded sufficiently, after which it was excited with a 280 nm UV diode. Figure 2b shows a series of emission spectra obtained during the ML experiment under a force of 24 N, by four movements of the rod pressed toward the sample plate. The SMSO sample clearly shows very intense, long‐lasting PersL, whose spectral shape does not differ from that of the PL spectrum (see Figure S1c, Supporting Information). The time shown on the *Z*‐axis was measured from the moment irradiation was terminated. The intensity of the emission band is clearly enhanced by the applied force; consequently, the emission spectrum should consist of two components: the PersL and ML bands, which are discussed later. Figure 2c shows how the integrated emission intensity, as calculated from a series of spectra acquired at various mechanical loading forces, varies with time. The sudden increase in intensity observed within the background of the diminishing PersL signal is caused by the motion of the rod across the sample and depends on the force exerted by the rod on the sample plate. To investigate how ML depends on mechanical loading, we determined the ML signal as an excess of the PersL signal acquired under the same conditions for each applied force listed in Figure 2c.

Using a mechanical loading of 24 N as an example, the PersL+ML (blue) curve in Figure 2d shows how the emission signal evolves with time, as calculated by integrating the peak area with the abscissa of the wavelength (in nm) for the spectra displayed in Figure 2b. The (green) PersL intensity curve was obtained in the same way and determined at subsequent time intervals but in the absence of mechanical loading. The PersL signal was subtracted from the PersL+ML signal to determine how ML intensity depends on time. The total ML signal induced by movements of the four rods across the sample plate was determined by integrating the ML signal over time. The integrated ML signal corresponds to the green area between the red and blue traces in Figure 2d. Figure 2e displays the resulting ML signal intensity of the SMSO powder as a function of applied force. The total ML intensity is linearly dependent on the applied force and increases with mechanical loading, resulting in an average sensitivity of 3.2%/N (the ML intensity at 30 N is denoted as 100%). Hence, our results clearly indicate that the developed sample can be used as an ML‐based optical stress sensor. Notably, in the case of ML stress sensing, sensitivity is typically characterized by the slope of the relative variation in the measured signal as stress increases. Higher sensitivity means a greater signal‐to‐noise ratio for a specific stress change, enabling the sensor to detect minor fluctuations in stress. However, the comparison of ML sensor performance is significantly limited by the absence of standardized measurement protocols, including setup and environmental conditions, as well as the diversity in microstructure and shape/form of the sensors. Please note that the ML intensity of most ML materials is linearly related to the applied stress, under a crucial prerequisite of operating below the elastic limit. Beyond the elastic range, the luminescence intensity reaches saturation. The degree of saturation depends on whether the stress can fully release the carriers necessary for luminescence. If the stress or impact is large enough, the luminescent center can be fully excited through energy transfer. For instance, if the ground state d‐electrons of Eu^2+^ ions are entirely excited by deformation, saturation will occur. The ML mechanism, including the relationship between light intensity and applied stress, is generally influenced by various factors, such as defects in the host materials, crystal structures, and the type and quantity of carriers. Many researchers have discovered such mechanisms theoretically^[^ ^55^ ^]^ and experimentally.″^[^ ^12^, ^56^ ^]^

Mechanical loading that affects the intensity of the emitted light accompanied by instantaneous and short‐term changes in the shape of its spectrum (i.e., PL‐PersL is stimulated by photons) is an essential feature of the ML phenomenon in the SMSO material. Specifically, a new broadband emission (ML) was observed with a maximum at ≈570–580 nm that partially overlapped with the PersL band; this observation was examined in more detail by performing the following ML experiments. The sample placed in the ML setup was maintained in the dark until the PersL signal had completely disappeared. The sample plate was then irradiated with UV light (280 nm) for 2 min and then maintained in the dark until the PersL signal had faded. ML was induced by pressing a glass rod at 40 mm s^−1^ against the sample plate with a force of 30 N. Figure 2f shows a series of spectra acquired 3 h after irradiation. ML was produced by pressing the rod against the sample plate with a force of 30 N and moving it four times. The glass rod was dragged across the sample plate four times, with a time gap of 4 s. Each pass of the rod generated a relatively weak emission signal in the 420–800 nm range. Despite the short sample‐irradiation duration, after 3 h of storage in the dark, the sample still exhibited a weak but measurable PersL signal, which is also evident in the upper trace shown in Figure 2g that displays the time dependence of the integrated emission signal. The green arrow indicates the moment that the slit of the spectrometer was opened. A similar experiment was carried out for a sample maintained in the dark for 5 h, the results of which are displayed as the lower trace in Figure 2g. The rod was moved ten times across the sample plate in 10‐s intervals (which corresponds to the 10 peaks shown in the lower trace) with the other measurement conditions unchanged. Although PersL faded to the level of the dark background signal, each rod movement still generated relatively weak short‐lasting ML and a broadband spectrum that peaked at ≈570 nm.

Figure 2h shows the normalized PersL spectrum (green) and spectra acquired under a mechanical load of 30 N: one was taken before the PersL signal had faded (blue) and is labeled PersL+ML, with the other acquired after the PersL signal had been quenched (red) and is labeled ML. Figure 2i shows the linear combination of the ML and PersL signals (cyan trace), which perfectly matches the acquired PersL+ML spectrum (blue). We conclude that the two components of the emission band are: I) the ML emission peak centered at ∼570–580 nm; and II) the PersL emission peak at ≈490 nm.

### 2.4 PL Thermometry

**Figure 3:**
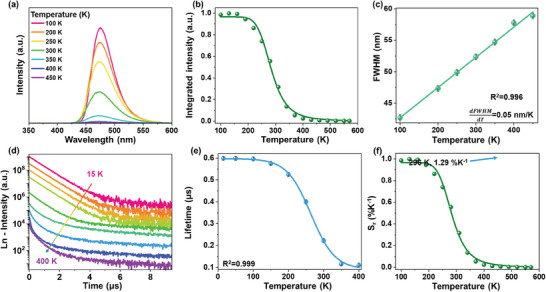
a) Thermal‐dependent PL emission spectra of SMSO when excited at 280 nm and b) corresponding integrated PL intensity as a function of temperature. c) FWHM as a function of temperature. d) Normalized temperature‐dependent decay profiles when excited at 290 nm. e) Lifetime as a function of temperature and f) corresponding lifetime‐mode‐based *S_r_* values.

We acquired temperature‐dependent PL spectra of the developed SMSO material within the 100–450 K range when excited at 280 nm (see **Figure** **3**a) to investigate how its PL properties respond thermally. The shapes and spectral positions of the bands underwent minimal change with increasing temperature. The integrated PL intensity is presented in Figure 3b as a function of temperature, which shows stability to 200 K above which a sudden decline is observed. The determined peak centroid as a function of temperature was fitted to equation (1), which includes a single de‐excitation process:

(1) $$I\;\left(T\right)=\frac{I_{0}}{1+A\exp\left(-\frac{E_{A}}{kT}\right)}$$

where *I_0_* is the PL intensity at 0 K, *E_A_* is the activation energy, and *A* is the relative probability of nonradiative de‐excitation, which led to *E_A_* = 2000 ± 100 cm^−1^ and *A *= 25 000 ± 1000. Figure 3c clearly shows that the full width at half maximum (FWHM) of the emission band increases linearly with increasing temperature (apparent thermal broadening of the band) (*R* ^2^ = 0.996). The sensitivity (d*FWHM*/d*T*) was determined to be ≈0.05 nm K^−1^ (≈1.25 cm^−1^ K^−1^).

Figure 3d displays the temperature‐dependent emission decay profiles of SMSO when excited at 290 nm in the 15–400 K temperature range. The decay profiles exhibit two distinct components: the first, within the 0–4 µs range, appears to decline with increasing temperature, while the second, which spans the 4–10 µs range, appears to remain constant across the entire temperature range. Each recorded decay curve is well fitted (*R* ^2^ > 0.99) to a bi‐exponential function that enabled the lifetime to be extracted:

(2) $$I\;=I_{0}\;+I_{1}e^{-\tau /t_{1}}+I_{2}e^{-\tau /t_{2}}$$

where *I_0_*, *I_1_*, *I_2_*, *t_1_*, and *t_2_* are fitting parameters, *I* is the intensity, and τ is time. As shown in Figure 3e, the determined lifetime tended to shorten, from 0.60 to 0.11 µs, as the temperature was increased from 15 to 400 K. The temperature dependence of the lifetime was well‐fitted to the Boltzmann function (*R* ^2^ > 0.999):

(3) $$\tau \;=A_{2}\;+\frac{A_{1}-A_{2}}{1+\exp\frac{T-T_{0}}{\sigma }}$$

with A_1_ = 0.598, A_2_ = 0.0916, T_0_ = 264.0 K, and σ = 33.9 K. Absolute sensitivity (*S_a_*) is typically used for both spectral‐shift‐ and FWHM‐based luminescence thermometry and manometry, as given by:

(4) $$S_{a}=\frac{dMP}{dT}\mathrm{or}\;\;S_{a}=\frac{dMP}{dP}$$

where MP is the measured parameter (e.g., bandshift or bandwidth (FWHM)), T is the temperature, and P is the pressure. Importantly, ratiometric and lifetime‐based thermometers generally use relative thermal sensitivity (*S_r_*) to describe their temperature‐ or pressure‐sensing performance, which can be expressed as follows:

(5) $$S_{r}=\;\frac{1}{MP}\times \frac{dMP}{dT}\;\mathrm{or}\;S_{r}=\;\frac{1}{MP}\times \frac{dMP}{dP}$$

with determined *S_r_* values shown in Figure 3f. Importantly, the synthesized sample exhibited a maximal relative sensitivity of 1.29%/K using lifetime as a thermometric parameter. Consequently, we demonstrated that the developed SMSO sensor, which thermally senses using two modes (i.e., FWHM‐ and lifetime‐based) can be used as a PL temperature gauge with satisfactory sensitivity. Please note, that in contrast to other sensing parameters, the lifetime‐based approach is insensitive to light scattering and reabsorption effects in different media, *i.e*., the effects which lead to erroneous temperature readouts in real‐world applications.^[^ ^48^, ^57^, ^58^ ^]^

### 2.5 PL Pressure Sensing

**Figure 4:**
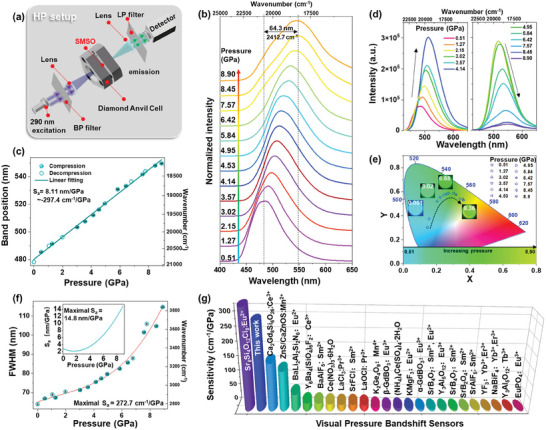
a) Simplified scheme of the HP measurement setup, showing the pressure chamber and optical geometry used. b) Normalized PL emission spectra of SMSO at various pressures recorded when excited at 290 nm, c) 4f^6^5d^1^–4f^7^ emission peak centroids as functions of pressure, and d) corresponding non‐normalized PL emission spectra. e) CIE diagram showing changes in emission color with pressure; the bottom panel shows PL color changes with pressure based on the CIE diagram; the inserted photographic images show PL colors at pressures of 0.001, 3.02, 6.68, and 9.36 GPa. f) The pressure‐dependent FWHM of the emission band; and the inset shows the corresponding absolute‐sensitivity profile. g) Pressure sensitivity (d*λ*/d*p*) of reported bandshift‐based visual pressure sensors.^[^ ^32^, ^35^, ^38^, ^54^, ^60^, ^61^, ^62^, ^63^, ^64^, ^65^, ^66^, ^67^, ^68^, ^69^, ^70^, ^71^, ^72^, ^73^, ^74^ ^]^ In all instances, the reported pressure sensitivity of a given sensor represents the highest attainable value for the respective pressure‐sensor material.

To investigate the potential application as an optical pressure gauge, we exploit the impact of pressure on the optical properties of the developed SMSO material. High‐pressure (HP) experiments were performed in a diamond anvil cell (DAC) at room temperature to a pressure of ≈10 GPa. A 290 nm UV diode was used as the excitation source (refer to the scheme in **Figure** **4**a that shows a simplified configuration of the HP measurement setup). HP experimental details are found in the SI. The pressure‐dependent normalized PL emission spectra (Figure 4b) show that the broad emission band attributable to the 4f^6^5d^1^–4f^7^ transition of Eu^2+^ undergoes a significant shift toward higher wavelength (redshift) with increasing pressure, with a 485.3 to 549.7 nm shift observed as the pressure was increased from 0.51 to 8.90 GPa, resulting in a total spectral displacement of ∼64.3 nm (i.e., an ≈2413 cm^−1^ lower energy difference between the ground and excited configurations). This observation is ascribable to the joint contributions of the pressure‐amplified nephelauxetic effect (reinforced bond covalency) and improved crystal field strength (enhanced splitting of the 4f^6^5d^1^ levels).^[^ ^32^, ^36^, ^37^ ^]^ Figure 4c reveals that the developed sensor shows a significant spectral shift that is linearly dependent on pressure (*R* ^2^ = 0.998), leading to an extremely high sensitivity of ≈8.11 nm/GPa (≈−297.4 cm^−1^/GPa), by using the peak emission‐band center as the manometric parameter. It should be noted that the determined S_a_ value (in wavenumbers, cm^−1^) is based on the bandshift and FWHM determined following the Jacobian transformation of the energy scale to minimize the readout error.^[^ ^59^ ^]^ The observed spectral shift was fully reversible during the compression–decompression process. According to the temperature‐dependent emission spectra shown in Figure 3a, the bandshift manometric parameter of the developed pressure sensor is highly temperature independent (i.e., d(*λ*)/d*T* = −0.0089 nm/K). The thermal invariability manometric factor (TIMF = S_r(P)_/S_r(T)_) of our pressure gauge was determined to be 911, consistent with good temperature invariance.^[^ ^37^ ^]^ Moreover, as shown in Figure 4d, the intensity of the emission band was observed to initially increase abnormally to ≈4.14 GPa, above which it tended to decline as the pressure was further elevated to 8.90 GPa. The enhanced emission intensity of the Eu^2+^ band is attributable to a shift in the absorption band with increasing pressure, leading to more efficient excitation pumping. The energy‐transfer process provides another possibility, as it is affected by the change in the energy of the 5d state associated with the charge‐transfer state, conduction‐band edge of the host lattice, and local defects.^[^ ^54^ ^]^ Figure 4e shows that the PL emission color is significantly tunable by changing the pressure, i.e., it changes from cyan to green and then finally to greenish‐yellow with increasing pressure, which indicates that the developed SMSO material can be used as a visual pressure sensor. The corresponding pressure‐dependent CIE chromaticity coordinates of the PL emission color are presented in Table S1 (Supporting Information). As shown in Figure 4f, the relationship between the determined FWHM and the pressure of the SMSO sample can be fitted to a third order polynomial function. The inset of Figure 4f presents the determined sensitivity as a function of pressure. It is evident that the FWHM mode of manometry can also achieve high sensitivity, ≈272.7 cm^−1^ GPa^−1^ (14.8 nm GPa^−1^), indicating the excellent pressure‐sensing performance of the sample.

Figure 4g displays the best pressure sensitivities with the aim of comparing the manometric performance of the developed SMSO sensor with that of other reported pressure gauges operating in the visible range.^[^ ^32^, ^60^, ^61^, ^62^, ^63^, ^64^, ^65^, ^66^, ^67^, ^68^, ^69^, ^70^, ^71^, ^72^, ^73^, ^74^ ^]^ The developed manometer exhibited a much higher sensitivity than almost all other reported visual HP sensors, with a sensitivity very close to that of the Sr_8_Si_4_O_12_Cl_8_:Eu^2+^ material, which was reported recently by our group.^[^ ^75^ ^]^ It should be noted that the SMSO sensor can function as a multifunctional sensing platform, thereby realizing the accurate, multi‐parameter simultaneous detection of temperature, pressure, and stress in ML, PL, and PersL modes. Such visual pressure sensors with ultrahigh sensitivities are particularly beneficial for detecting pressure in heavy construction facilities, quality inspection, and building collapse disaster control using simple devices, such as UV diodes/lamps, and naked‐eye observations.

### 2.6 PersL‐Lifetime‐Based Pressure Sensing

**Figure 5:**
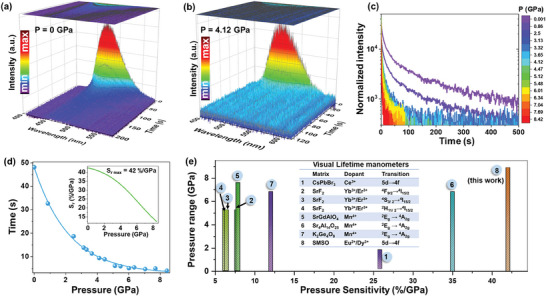
Exemplary time‐resolved afterglow emission spectra (recorded every 0.5 s) under a) ambient conditions and b) at 4.12 GPa. c) Afterglow (PersL) decay curves based on the corresponding time‐resolved emission spectra. d) Determined afterglow lifetime as a function of pressure; the inset shows the corresponding lifetime‐mode relative pressure sensitivity. e) Pressure sensitivities (d*λ*/d*p*) of reported lifetime‐based luminescent manometers.^[^ ^76^, ^77^, ^78^, ^79^, ^80^, ^81^, ^82^ ^]^

Compressing a material can also change the charge‐transfer rate between Dy^3+^ and Eu^2+^, which is manifested as altered luminescence decay profiles and lifetimes. Time‐resolved afterglow emission spectra were recorded as a function of pressure, and selected spectra acquired at atmospheric pressure and 4.12 GPa are shown in **Figures** **5**a,b, respectively. Noise is much more prominent in the latter case owing to pressure‐induced quenching of the PersL signal, which is detrimental to sensing accuracy at higher pressures.^[^ ^37^, ^48^ ^]^ Interestingly, the recorded PersL decay curves were observed to change significantly with increasing pressure (Figure 5c). The determined lifetime shows a strong pressure dependence and shortens from 48.2 s at atmospheric pressure to 4.02 s at 8.42 GPa (Figure 5d). This long lifetime is ascribable to the thermally stimulated and delayed release of the excitation energy stored in the energy traps of the SMSO particles. The other potential factors influencing the lifetime could be the depth of the traps and the transport of electrons to the luminescent centers.^[^ ^37^, ^76^, ^77^, ^78^, ^79^ ^]^ As shown in the inset of Figure 5d, the determined lifetime‐based relative pressure sensitivity (S_r_) decreases with increasing pressure, with a maximum S_r_ of 42%GPa^−1^ recorded under ambient conditions; it is worth noting that such a high S_r_ value is rarely observed. Moreover, this is the first report that demonstrates the pressure‐sensing use of PersL and its long‐lasting (seconds) lifetime as a manometric parameter. To compare the pressure‐sensing performance of the SMSO material with other reported lifetime‐based luminescence manometers, Figure 5e shows the pressure sensitivities and pressure ranges of various manometers.^[^ ^76^, ^77^, ^78^, ^79^, ^80^, ^81^, ^82^ ^]^ The developed SMSO manometer is clearly the most sensitive among reported visual and luminescent HP sensors, which confirms that the developed SMSO material can be used as a bimodal pressure gauge with superior sensitivity. Consequently, facile readouts are facilitated owing to the long‐lasting PersL lifetimes of the material, which eliminates the need for sophisticated and expensive pulsed excitation sources and fast detectors/electronics.

It is worth noting, that concerning the limitations and conditions for the observed phenomena, the obtained upper limits for force were 30 N, for temperature 400 K, while in the case of pressure, they were up to 9 GPa. Both temperature and pressure are the actual working limits because the luminescence intensity is too weak above these values to give reliable results. In the case of force, this limit is undoubtedly higher than 30 N, and the measurement limitation results from the mechano‐optical setup used, which could be improved.

### 2.7 3D‐Printing and Optical Coding

**Figure 6:**
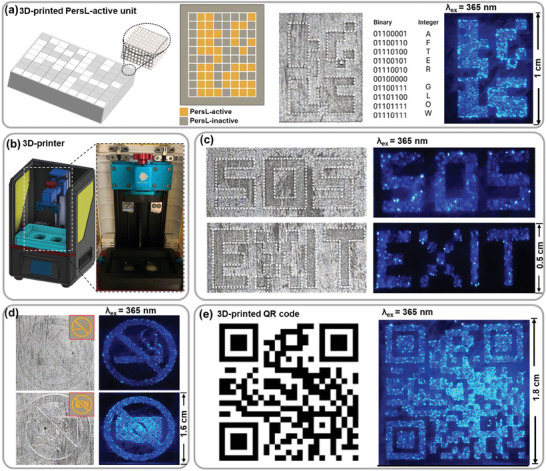
Demonstrating anti‐counterfeiting, night‐vision safety‐sign, 8‐bit optical‐coding, and QR‐code applications using 3D‐printed polymers containing SMSO particles that exhibit efficient PersL. a) Binary 8 × 10 matrix with encoded information (“AFTER GLOW”) using PersL‐inactive units as “0” and PersL‐active units as “1.” b) The 3D‐printing device is used to print the PersL‐active polymer. 3D‐printed PersL signs showing c) “SOS” and “EXIT” and d) “no smoking” and “no photos” images. e) Designed QR pattern and the corresponding optical image of the QR code for information encryption prepared using SMSO particles. All PersL photographic images were acquired after excitation with a 365 nm UV lamp in the dark (UV off).

To showcase the potential applications of the developed SMSO material, anti‐counterfeiting, night‐vision safety‐sign, full optical‐coding, and QR‐code multifunctional platforms were fabricated using a 3D‐printable polymer and a 3D‐printing device, as shown in **Figure** **6**a–d. We first used the developed SMSO material to demonstrate an advanced, power‐free, luminescence‐based information‐storage application using high‐resolution (≈50 µm) 3D‐printing technology to fabricate desired patterns. We initially 3D‐printed an 8 × 10 (250 × 250 µm) matrix composed of SMSO particles embedded in the polymer (Figure 6a), with optical information encoded in the form of binary codes. The prestored information was revealed by transforming the PersL signals into an 8‐bit ASCII code. In the patterns, the values of “0” are represented by optically inactive polymer units, while PersL‐active polymer units correspond to “1.” Therefore, the hidden information (“AFTER GLOW”) was revealed in the encrypted pattern after signal decoding. Figure 6b shows a simplified scheme and a photographic image of the 3D printer. As shown in Figure 6c, the letters were fabricated by embedding the SMSO material in a polymer followed by 3D‐printing, with subsequent exposure to UV light for 2 min, followed by storage in the dark. The righthand part of Figure 6c shows that “SOS” and “EXIT” are revealed in PersL mode. Similarly, two graphical patterns that reveal “no smoking” and “no photos” signs were 3D‐printed and are clearly observable (after UV irradiation) in the dark without excitation (UV off), as shown in Figure 6d. Such PersL materials combined with 3D‐printing technology are expected to be beneficial in power‐outage cases and when personnel need to be evacuated in emergencies, as well as in energy‐saving and environmental‐protection applications. Finally, we present a 3D‐printed quick response (QR) code using a polymer matrix composed of the SMSO material that emits a glaringly blue signal (Figure 6e). A smartphone is redirected to the website of our faculty (chemia.amu.edu.pl) when the QR code is scanned in a dark environment. It is worth noting, that there are no issues with the long‐term information storage using our material, thanks to its great structural and optical stability. Moreover, the distribution of SMSO particles within the polymer matrix is relatively uniform, as shown in PersL images. However, the brighter spots observed in the photographs indicate some aggregation of the particles in the 3D‐printed patterns, which in fact is practically inevitable during the fabrication process with micron‐sized particles of irregular shape.

## 3 Conclusion

Herein, we presented a versatile optical‐sensing platform that exploits three distinct types of luminescence: PL, PersL, and ML. This platform is based on SMSO (Sr_2_MgSi_2_O_7_:Eu^2+^/Dy^3+^) and facilitates the precise detection of temperature, pressure, and stress. Our SMSO material works as a sensitive mechanoluminescent stress sensor that accurately measures forces within the 3–30 N range (at least) while demonstrating linearity between ML intensity and applied force. Furthermore, the FWHM and PL lifetime of this material are notably sensitive to temperature fluctuations, with values of ≈0.05 nm K^−1^ and 1.29%K^−1^, respectively. Additionally, the investigated SMSO material can operate as a dual‐mode pressure sensor with outstanding sensitivity, serving as a persistent luminescence lifetime manometer (reported for the first time) with a sensitivity of ≈−297.4 cm^−1^ GPa^−1^, and as a PL bandshift manometer with a relative sensitivity (S_r_) of 42%GPa^−1^. Remarkably, the material can visually detect high pressures owing to pronounced changes in its photoluminescence color, in which very large pressure‐induced changes are clearly visible to the naked eye. Finally, the prepared material was combined with 3D‐printing technology, and anti‐counterfeiting, optical‐coding, QR‐code, and night‐vision applications were demonstrated using a polymer embedded with optically active SMSO particles. This work also provides some guidelines for future research to develop resemble multifunctional materials with better ML performance and broader working ranges for temperature, pressure, and force.

## 4 Experimental Section

Detailed experimental procedures are provided in the Supporting Information.

## Conflict of Interest

The authors declare no conflict of interest

## Supporting information



Supporting Information

## Data Availability

The data that support the findings of this study are openly available in [Zenodo] at [https://doi.org/10.5281/zenodo.12726717], reference number [12726717].
