# Supplementary material for: Persistent Photoluminescence and Mechanoluminescence of a Highly Sensitive Pressure and Temperature Gauge in Combination with a 3D‐Printable Optical Coding Platform
Source: Adv Sci (Weinh). 2024 Oct 10;11(44):2408686. doi: 10.1002/advs.202408686 (PMC11600286; doi:10.1002/advs.202408686)
Supplement: Supplementary file 1 — Supporting Information [file ADVS-11-2408686-s001.docx]

Supporting information for

**Persistent Photoluminescence and Mechanoluminescence of a Highly Sensitive Pressure and Temperature Gauge in Combination with a 3D-printable Optical Coding Platform**

*Teng Zheng, Jiangcheng Luo, Dengfeng Peng,* Liang Peng,* Przemysław Woźny, Justyna Barzowska, Mikołaj Kamiński, Sebastian Mahlik, Jan Moszczyński, Kevin Soler-Carracedo, Fernando Rivera-López, Hanoch Hemmerich, Marcin Runowski,**

Dr. T. Zheng, **Prof. L. Peng**

School of Information and Electrical Engineering,

Hangzhou City University

Hangzhou 310015, China

E-mail: **pengl@hzcu.edu.cn**

Dr. J. Luo,

Key Laboratory of Optoelectronic Devices and Systems of Ministry of Education and Guangdong Province

College of Physics and Optoelectronic Engineering

Shenzhen University

Shenzhen 518060, China

**Prof. D. Peng**

Shenzhen Key Laboratory of Intelligent Optical Measurement and Detection;

Key Laboratory of Optoelectronic Devices and Systems of Ministry of Education and Guangdong Province, College of Physics and Optoelectronic Engineering;

Shenzhen Key Laboratory of Photonics and Biophotonics,

Shenzhen University, Shenzhen, China

Shenzhen 518060, China

E-mail: **pengdengfeng@szu.edu.cn**

Dr. P. Woźny, J. Moszczyński, Dr. K. Soler-Carracedo, **Prof. M. Runowski**,

Faculty of Chemistry

Adam Mickiewicz University

Uniwersytetu Poznańskiego 8

Poznań 61-614, Poland

E-mail: **runowski@amu.edu.pl**

Dr. J. Barzowska, M. Kamiński, Prof. S. Mahlik,

Institute of Experimental Physics

Faculty of Mathematics, Physics and Informatics,

Wita Stwosza 57

University of Gdansk,

Gdansk 80-308, Poland

Dr. K. Soler-Carracedo, H. Hemmerich

Departamento de Física, IUdEA, IMN and MALTA Consolider Team,

Universidad de La Laguna

San Cristóbal de La Laguna E-38200, Santa Cruz de Tenerife, Spain

Prof. F. Rivera-López

Departamento de Ingeniería Industrial

Escuela Superior de Ingeniería y Tecnología

Universidad de La Laguna

San Cristóbal de La Laguna E-38200, Santa Cruz de Tenerife, Spain

**Experimental Section**

***Synthesis***

High-quality raw materials, i.e., SrCO_3_ (>99%, Sinopharm Co., Ltd.), MgO (>99%, Sigma-Aldrich), SiO_2_ (99.99%, Aladdin), Eu_2_O_3_ (99.99%, Sinopharm Co., Ltd.), and DyCl_3_ × 6H_2_O (99.99%, Sinopharm Co., Ltd.) were used for the synthesis of the SMSO: 1%Eu^2+^, 0.25%Dy^3+^, SMSO: 2%Eu^2+^, 0.25%Dy^3+^ and SMSO: 2%Eu^2+^, 2%Dy^3+^ materials. The mixture was transferred into an alumina crucible and then placed in a tube furnace. Then, it went through a heating process of 1400 ℃ for 5 hours under a reducing gas mixture (N_2_/H_2_ = 95%/5%). After annealing, the samples were cooled to room temperature and grounded for further characterization.

***3D-printing processs of SMSO: 2%Eu^2+^, 2%Dy^3+^ doped polymer***

For 3D-printing, the raw polymer was a Simple Siraya Tech UV resin, consisting in a mixture of urethane acrylate (CAS No. 877072-28-1, wt% = 20–50%), acrylic monomer (CAS No. 64401-02-1, wt% = 30–60%), and photoinitiator (CAS No. 119-61-9, wt% = 0–5%). This polymer was doped with a 10 wt.% of Sr_2_MgSi_2_O_7_: Eu^2+^, Dy^3+^ material. The 3D prototypes were previously designed using Fusion 360, and the digital file was introduced in Lychee Slicer software for the selection of the printing parameters. From the final model, the anti-counterfeiting, night-vision safety signs, 8-bit optical coding, and QR code were printed by Stereolithography using an Anycubic Photon printer. After printing, the pieces were cleaned with a 99.9% isopropyl alcohol.

1. ***Photoluminescence characterization***

***Ambient.*** Ambient photoluminescence characterization was carried out in an Edinburgh Instruments Spectrometer FLS 1000.

***High temperature.*** Light-emitting diode (LED) emitting at a maximum wavelength of 280 nm served as the excitation source for temperature-dependent measurements. Temperature control within the range of 100 – 600 K was achieved using the THMS600 Linkam stage temperature controller in conjunction with the LNP95 liquid nitrogen cooling pump system. An Andor SR-750-D1 spectrometer, equipped with a CCD camera (DU420A-OE) and operating in the 350–1000 nm wavelength range, was employed to measure the temperature-dependent emission spectra. Time-resolved luminescence spectra were acquired by exciting the sample using a pulsed PG 401/SH optical parametric generator with tunable wavelength, which was pumped by a PL2251A pulsed YAG:Nd laser from EKSPLA. The emitted signal was detected by a C4334-01 streak camera (Hamamatsu) coupled with a 2501S grating spectrometer from Bruker Optics.

***High pressure.*** High-pressure measurements were done in a diamond anvil cell (DAC) with a mixture of methanol-ethanol-water (volume ratio of 16:4:1) as a pressure-transmitting medium and ruby (Al_2_O_3_:Cr^3+^) as a pressure inhibitor. Emission spectra were recorded using an Andor Shamrock 500i spectrometer with an iDus CCD camera as a detector and the 280 nm UV LED as the excitation source. The recorded spectra were corrected for the apparatus response. Persistent luminescence was also recorded at high pressure in DAC. Due to long persistent luminescence, the emission spectra were recorded in the kinetic mode recording emission spectra in 15 ms intervals, luminescence intensity was integrated and correlated with the time of experiments.

1. ***Persistent-luminescence characterization.***

280 nm UV LED was used as an excitation source, and the signal was detected using an Andor Shamrock 500i spectrometer with an iDus CCD camera detector. The recorded spectra were corrected to account for the apparatus response.

1. ***Mechanoluminescence characterization.***

The custom-built setup controlled by custom-made software in the LabView environment was used to measure friction-induced mechanoluminescence (ML). For the ML experiments, a layer of evenly spread powdered sample was fixed to the PMMA plate (0.3 mg/mm^2^) with specially selected adhesive tape. The glass rod mounted on a linear rail was dragged across the sample plate with a set force, speed, and frequency to induce ML. The ML signal was collected using a Shamrock 500i spectrometer with TEC camera iDus420 (Andor Technology). Please note that before the PersL and ML experiments, the sample plate was placed in the F-ML setup and was kept in the dark until the PersL signal faded enough, namely, the PersL spectrum reached at its maximum level not more than 5 counts/60 ms above the dark background which was 1 ± 2 counts/60 ms. After that, the sample was exposed to a 280 nm UV diode for 2 minutes. As shown in Figure 1, to induce ML, the glass rod pressed against the sample plate with a specified force ranging from 3 to 30 N, was dragged across the sample with a speed of 40 mm/s, four times every 4 s. For each force value, the rod started its first of four movements 30 s after the end of the sample irradiation stage. The emission spectra were collected every 60 ms.


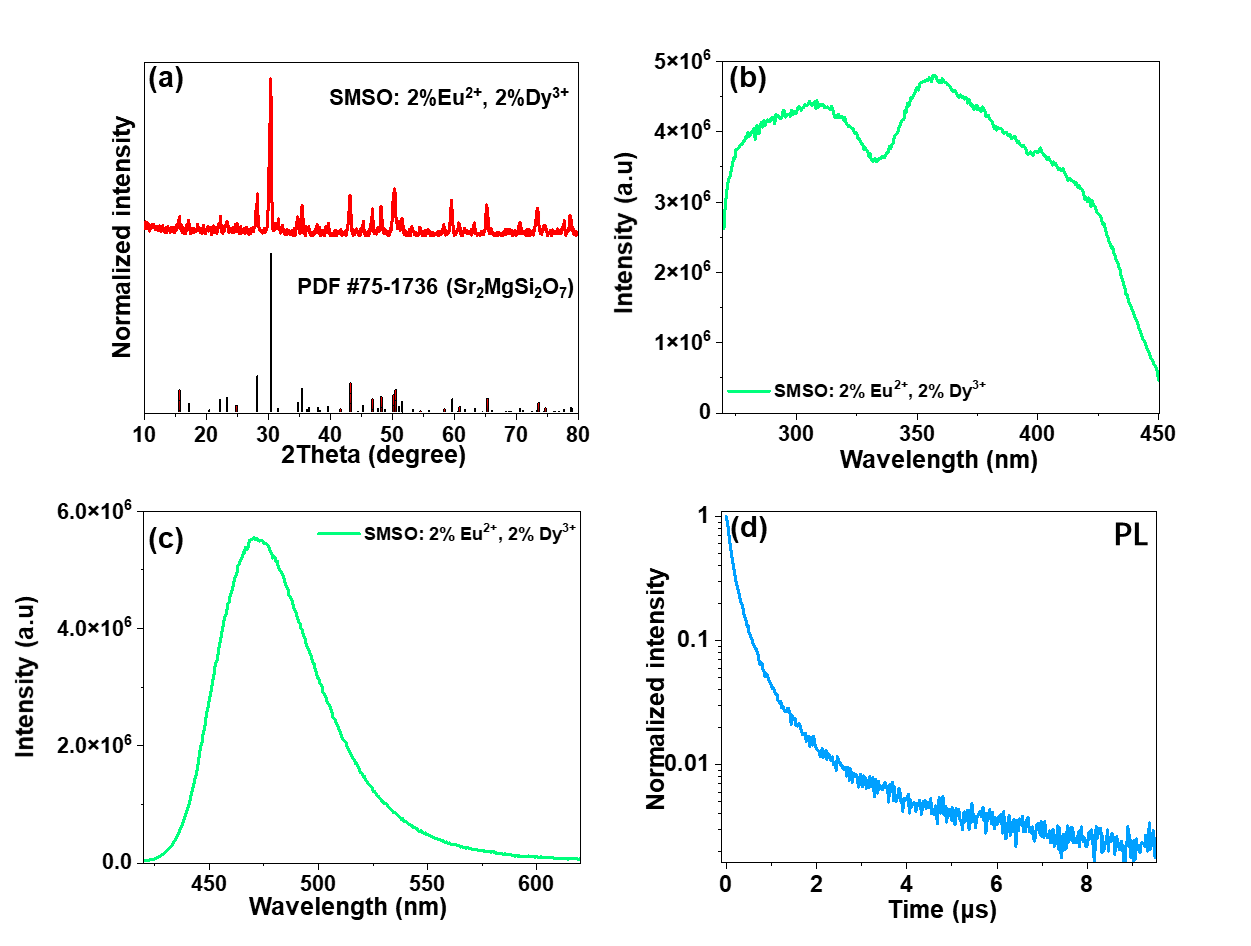


**Figure S1**. (a) The XRD patterns of the synthesized SMSO: 2%Eu^2+^, 2%Dy^3+^ material. (b-c) The corresponding PL excitation and emission spectra. (d) The corresponding decay curve of the sample.


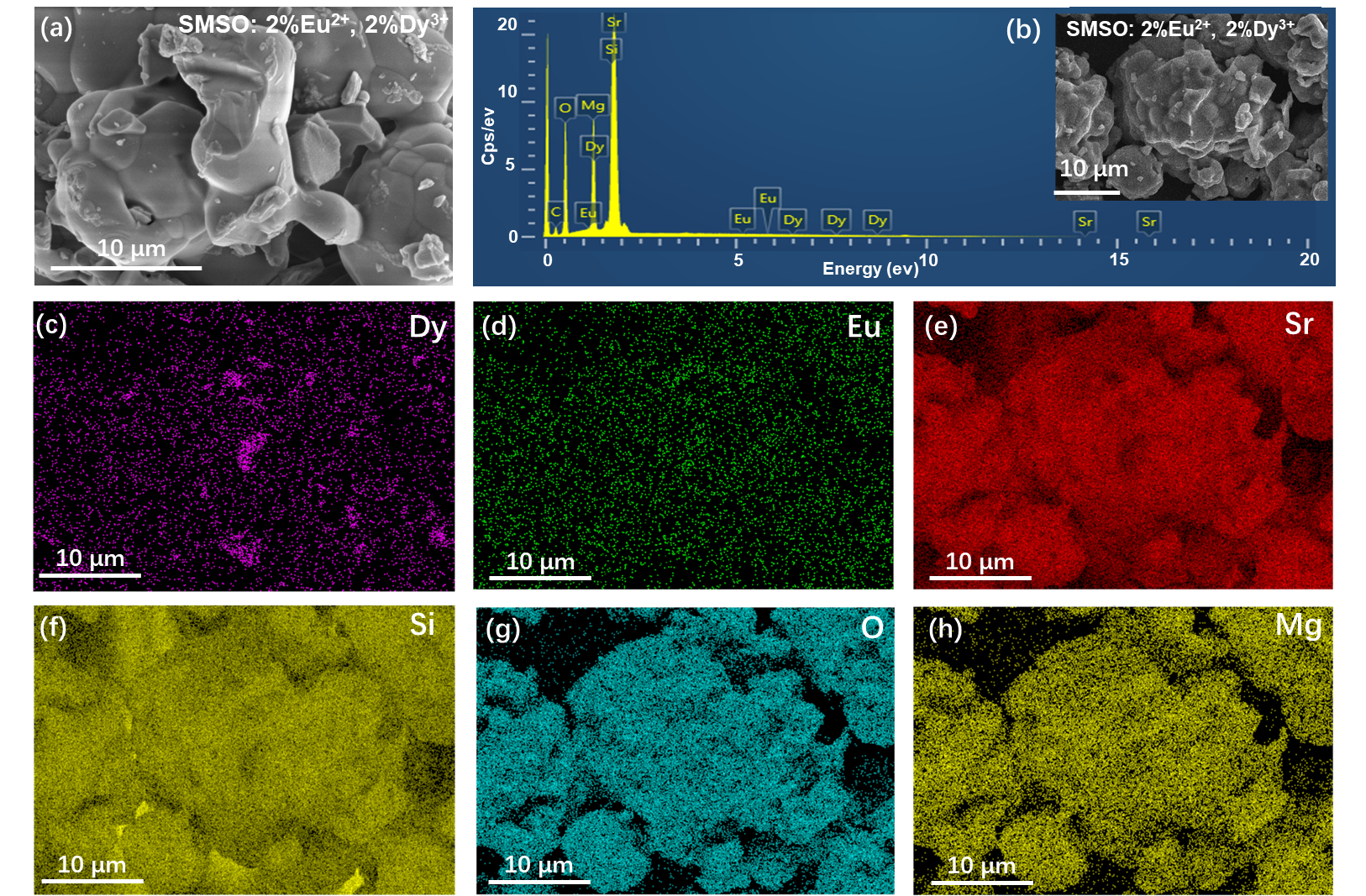


**Figure S2**. (a) The SEM images of the synthesized samples as a function of dopant content. (b) The EDX spectra. The inset shows the EDX layered image of the selected SMSO: 2%Eu^2+^, 2%Dy^3+^ sample. (c) The elemental mapping of Dy, Eu, Sr, Si, O, and Mg elements in the sample.


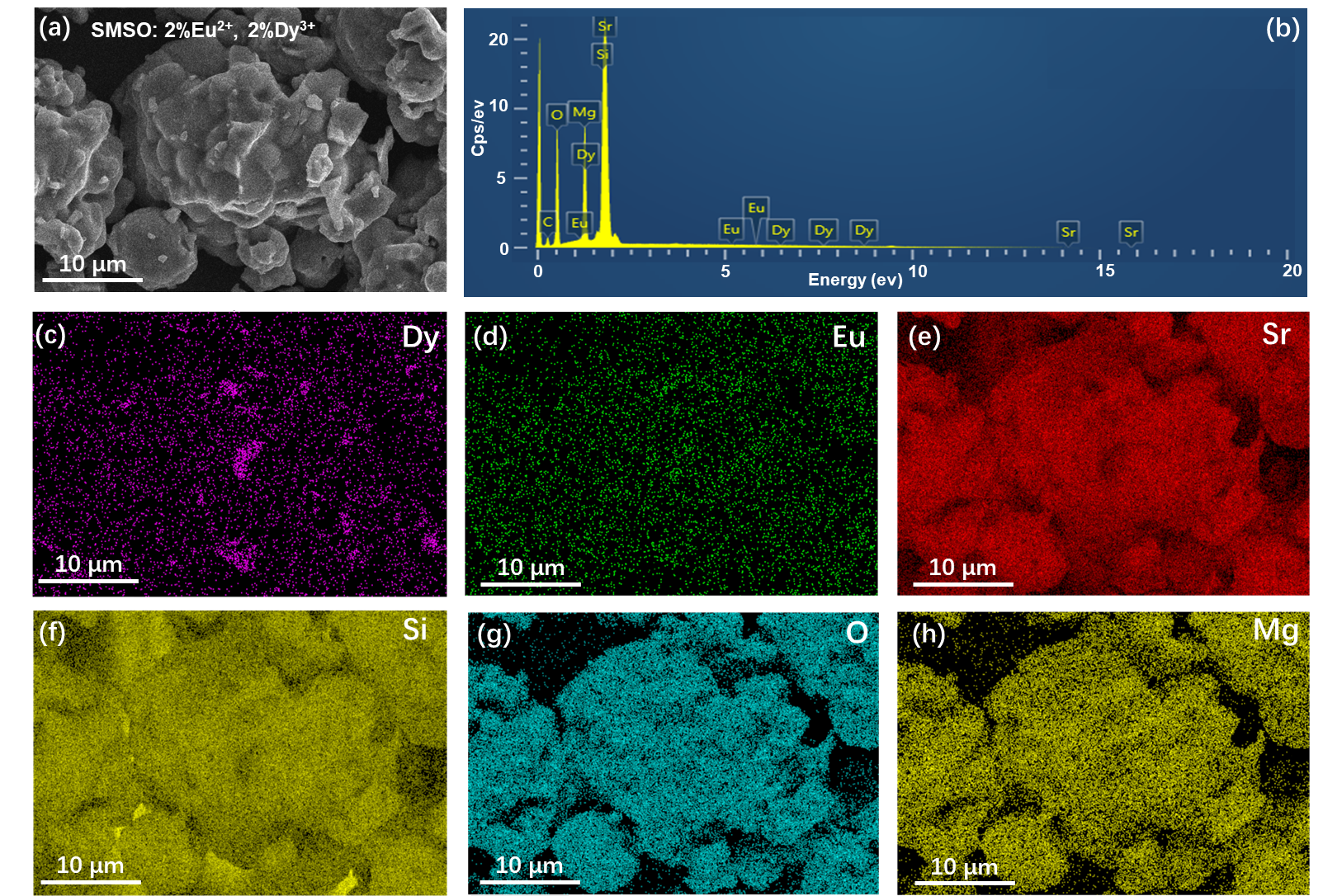


**Table S1.** The CIE chromaticity coordinates of the PL emission color as a function of pressure of the developed visual pressure sensor.

| Pressure (GPa) | X | Y |
| --- | --- | --- |
| 0.51 | 0.14427 | 0.30219 |
| 1.27 | 0.15598 | 0.3731 |
| 3.02 | 0.20515 | 0.52007 |
| 3.57 | 0.22426 | 0.5496 |
| 4.14 | 0.24489 | 0.56858 |
| 4.53 | 0.26002 | 0.57826 |
| 4.95 | 0.27601 | 0.58376 |
| 5.84 | 0.30681 | 0.58272 |
| 6.42 | 0.32206 | 0.571 |
| 7.57 | 0.3464 | 0.53752 |
| 8.45 | 0.36477 | 0.53293 |
| 8.9 | 0.36909 | 0.52403 |
